# A-site compositional effects in Ga-doped hollandite materials of the form Ba_x_Cs_y_Ga_2x+y_Ti_8−2x−y_O_16_: implications for Cs immobilization in crystalline ceramic waste forms

**DOI:** 10.1038/srep27412

**Published:** 2016-06-07

**Authors:** Yun Xu, Yi Wen, Rob Grote, Jake Amoroso, Lindsay Shuller Nickles, Kyle S. Brinkman

**Affiliations:** 1 Department of Materials Science and Engineering, Clemson, SC, USA; 2 Department of Environmental Engineering and Earth Science, Clemson, SC, USA; 3 Savannah River National Laboratory, Aiken, SC, USA

## Abstract

The hollandite structure is a promising crystalline host for Cs immobilization. A series of Ga-doped hollandite Ba_x_Cs_y_Ga_2x+y_Ti_8−2x−y_O_16_ (x = 0, 0.667, 1.04, 1.33; y = 1.33, 0.667, 0.24, 0) was synthesized through a solid oxide reaction method resulting in a tetragonal hollandite structure (space group *I4/m*). The lattice parameter associated with the tunnel dimension was found to increases as Cs substitution in the tunnel increased. A direct investigation of cation mobility in tunnels using electrochemical impedance spectroscopy was conducted to evaluate the ability of the hollandite structure to immobilize cations over a wide compositional range. Hollandite with the largest tunnel size and highest aspect ratio grain morphology resulting in rod-like microstructural features exhibited the highest ionic conductivity. The results indicate that grain size and optimized Cs stoichiometry control cation motion and by extension, the propensity for Cs release from hollandite.

One of the major challenges confronting the nuclear power industry is to provide an enduring solution to the problem of high-level waste disposal^1^. Cesium is one of the more problematic fission product radionuclides to immobilize because of its volatility at high temperature and its tendency to form water-soluble compounds. One strategy to immobilize Cs is incorporation into borosilicate glass^2^. However, the limited waste loading and low leach-resistance of borosilicate glass has motivated the search for an improved waste form with a higher waste loading capacity and dramatically improved resistance to elemental release^3^,^4^. Ceramic waste forms have been studied due to their superior chemical durability and the ability to incorporate a broad spectrum of chemical species within the available lattice sites^5^,^6^. A number of crystalline ceramic materials such as apatite, pollucite and titanate-based compounds, such as hollandite, have been explored for the immobilization of Cs^7^. Hollandite-type compounds, generally expressed as A_x_M_8_O_16_, are widely proposed candidates to host and immobilize Cs in a crystalline waste form. Hollandites are actually a subset of minerals in the priderite group, (K,Ba)(Ti,Fe)_8_O_16_, in which the majority of the M-site is replaced with Ti. Illustrated in Fig. 1, hollandite-type structures can exist as both tetragonal (*I4/m*) and monoclinic (*I2/m*) phases, exhibiting characteristic tunnels parallel to the unique axis. These tunnels are constricted periodically by four oxygen (O^2−^) ions that form a bottle-neck, and which act to impede the mobile alkali and alkaline-earth ions that reside on the A-site in these tunnels^10^. A-site cations occupy tunnel sites formed by octahedrally-coordinated M-site cations. The structure can host a range of cation substitution possibilities, such as K^+^, Na^+^, Cs^+^, Ba^2+^, and Rb^2+^ on the A-site^11^,^12^,^13^ and Fe^2+/3+^, Al^3+^, Ga^3+^, and Ti^4+^ on M-site. A number of studies have been conducted on how the M-site dopant affects microstructure and Cs incorporation^14^. Among those cations studied (Al^3+^, Cr^3+^, Ga^3+^, Fe^3+^), Ga^3+^ demonstrates the ability to lower the melting point of synthetic hollandite and provide redox stability, which is of interest to current US DOE efforts in waste forms aimed at melt processing^15^,^16^,^17^,^18^.

Historically, hollandite-type structures have been widely studied for their potential as fast-ionic conductors due to the high mobility of A-site cations in tunnels^19^,^20^. The relatively high cation mobility, characteristic of these structures, is counterintuitive to their application as a waste form. Under the driving forces imposed from local chemical concentration gradients and heat generated by radioactive decay, large cation mobility would enhance the accumulation of Cs at grain boundaries and exposed surfaces, where corrosion and leaching is the most significant. Therefore, the mobility of the cations (Cs) should be as small as possible for waste form applications. There have been limited studies to date of the complex impedance of Ba-hollandite and Cs-incorporated hollandite. Ba- or Cs-hollandites were found to have low tunnel cation mobility and have the potential to be a stable waste form^21^,^22^. Those impedance studies were limited to baseline compositions with a fixed A-site stoichiometry of Ba_1.04_Cs_0.24_, which has been the established SYNROC composition used in ceramic waste forms for several decades. In fact, little work has been done regarding the propensity for phase formation and cation mobility in tunnel sites as a function of A-site stoichiometry. This work aims to fill this gap by examining structure, phase formation, and conductivity of the hollandite series Ba_x_Cs_1−x_ Ga_1+x_ Ti_7−x_O_16_.

In this paper, the atomic- and micro- structural effects on the mobility of Ba and Cs in the hollandite structure were determined and the implications of these results for waste form applications is discussed. Specifically, a solid-solution series of hollandite Ba_x_Cs_y_Ga_2x+y_Ti_8−2x−y_O_16_ (x = 1.33, 1.04, 0.667, 0; y = 0, 0.24, 0.667, 1.33) was fabricated and characterized. These samples are referred to as Ba1.33, Ba1.04Cs0.24, Ba0.667Cs0.667, Cs1.33. The compositions (Ba_x_Cs_y_Ga_2x+y_Ti_8−2x−y_O_16_ (x = 1.33, 0.667, 0; y = 0, 0.667, 1.33) were derived in a way to achieve the lowest computational cost for density functional theory (DFT) calculations. The detailed design methodology is described in experimental section. In addition, a baseline hollandite composition Ba1.04Cs0.24 was included to compare the results with previous studies.

## Results

### Final enthalpy of formation

The enthalpy of formation based on the simple oxides were computed from DFT calculations. The formation enthalpies of Ba1.33, Ba0.67Cs0.67, and Cs1.33 are listed in Table 1. The negative formation enthalpies indicated that all compositions are thermodynamically favorable, with the cesium end-member Cs1.33 representing the most energetically favorable composition. These calculated enthalpies are similar to experimentally determined values for formation enthalpies measured by Costa using melt solution calorimetry^23^.

### Crystal structure

X-ray diffraction confirmed that all samples exhibited primarily hollandite phases with the exception of a minor peak near 28 degrees 2θ as indicated by the asterisk in Fig. 2a. This peak is presumed to be associated with a Ti-rich phase devoid of Cs that was identified by detailed SEM-EDX analysis. All samples were found to have a tetragonal structure (*I4/m*). Figure 2a displays the shift in the (301) reflection peak with increasing Cs concentration, which indicates lattice distortions resulting from an increase in the tunnel diameter. The framework ions (M-sites) and the size and concentration of the mobile ions (A-sites) affect the tunnel diameter and consequently the lattice parameter. In particular, larger A-site ions, such as the cesium ion (Cs^+^), expand the tunnel diameter and lattice parameter. Rietveld refinements and DFT calculations confirmed an increase of *a* lattice parameter with increasing Cs concentration (Fig. 2b) with negligible changes to the c lattice parameter. Increased Cs content resulted in negligible changes to the *c* lattice parameter^23^. In this study, as the Cs content was increased, the Ga concentration was decreased in order to maintain charge neutrality in the material. A decreased Ga concentration results in a smaller average M-site dopant ionic radius. Thus, larger A-site ion substitutions are expected to have a greater effect on the lattice parameter than distortions due to framework ion substitutions.

Back-scattered electron images showing the microstructure and morphology of the reacted pellets are shown in Fig. 3. All samples crystallized with a rod-like morphology, typical of hollandite^24^. A microstructural feature of interest was the observation of increased rod length with increasing Cs concentration. The homogeneity of the samples was confirmed using contrast imaging and chemical analysis in low magnification images taken at a variety of locations throughout the sample. Table 2 lists the target compositions and the calculated compositions determined from EDX analysis and ICP chemical analysis. A comparison of the EDX determined composition and the ICP measured compositions indicate close agreement between the two techniques. In general, the results confirm that the targeted stoichiometries were obtained. Literature studies on the baseline composition Ba_1.04_Cs_0.24_Ga_2.32_Ti_5.68_O_16_ (Ba1.04Cs0.24) frequently indicated significant Cs deficiency in the sintered samples^2^. In this study, we obtained 88.3% Cs retention according to the ICP analysis, which is much higher than the 50% retention typically reported due to Cs volatility during high temperature sintering^14^. The higher Cs retention is attributed to the use of a controlled atmosphere during synthesis that resulted in a high Cs vapor pressure in the system, reducing the driving force for Cs loss from solid to vapor phase. In addition, relative densities shown in Table 2 demonstrate these samples are fairly dense. Decreasing relative density from Ba1.33 to Cs1.33 is possibly due to the Cs volatilization during heating process.

### Ba/Cs ionic mobility

**Figure 1:**
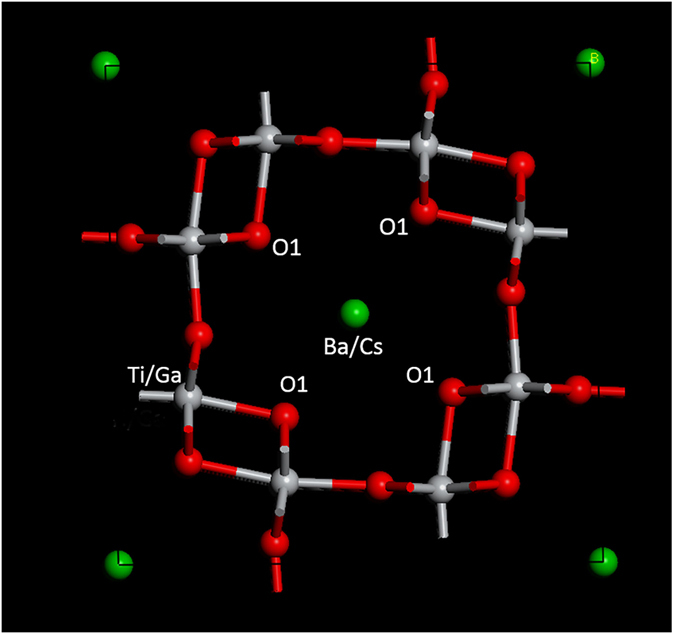
Perspective view of hollandite along [001] direction.

**Figure 2:**
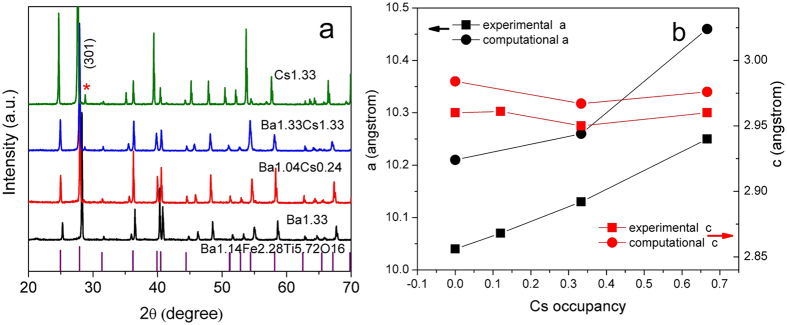
(**a**) XRD patterns of the synthesized hollandite containing different Cs levels with fixed A site occupancy of 1.33/2. *Indicates secondary phase detected. (**b)** Evolution of lattice parameters versus Cs content: black lines and points are lattice parameter a; red lines and points are lattice parameter c.

**Figure 3:**
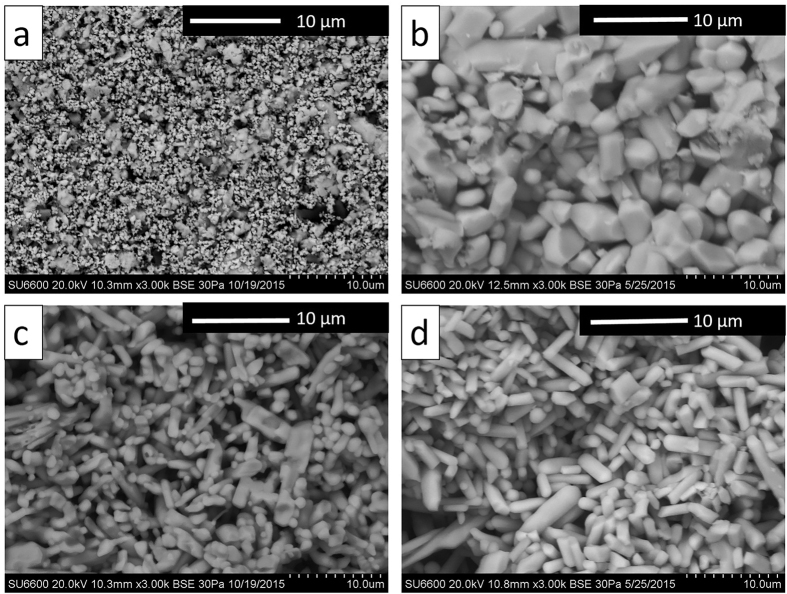
SEM-Backscattered Electron micrograph of the sintered pallets at magnification of 300X, (**a**) Ba_1.33_Cs_0_Ga_2.32_Ti_5.68_O_16_; (**b**) Ba_1.04_Cs_0.24_Ga_2.32_Ti_5.68_O_16_; (**c**) Ba_0.667_Cs_0.667_Ga_2_Ti_6_O_16_. (**d**) Cs_1.33_Ga_1.33_Ti_6.67_O_16_.

**Figure 4:**
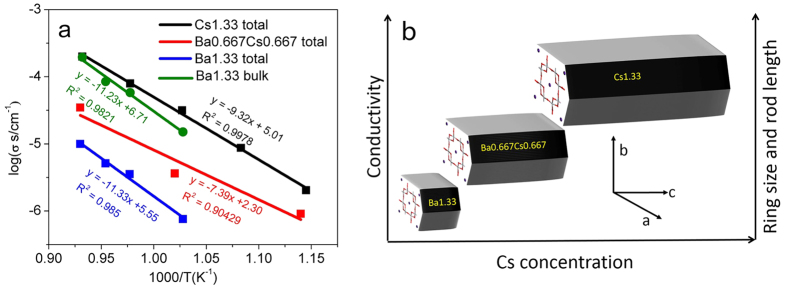
(**a**) Arrhenius plots of hollandite with different Cs doping levels. Scattered points are experimental data, lines are linear fitted data. (**b)** Graphic depicting the variation of ionic conductivity in the hollandite structure as a function of composition (Cs concentration). Increasing Cs concentration impacts atomic structure through ring size expansion and microstructure by formation of rod-like features, which grow along c axis; both of these effects lead to an increase in conductivity with increasing Cs concentration.

Ba/Cs ionic mobility is thought to be an important indicator of the suitability of hollandite to immobilize reprocessed nuclear waste. Complex impedance measurements of sintered hollandite with the composition Ba_1_Cs_0.28_Fe_0.82_Al_1.46_Ti_5.72_O_16_ displayed low conductivity values on the order of 10^−7^ (S.cm^−1^) at 800 °C. The low conductivity was attributed to the low mobility of Cs^+^ in the hollandite tunnels. In addition, the presence of barium ions (Ba^2+^) created the bottleneck region of linked oxygen anions that is showed schematically in Fig. 1 25. Other work on related systems by Aubin Chevaldonnet *et al.* found that a similar hollandite system, Ba_1.16_Al_2.32_Ti_5.68_O_16_, exhibited an ionic conductivity of 4.4^−8^ (S.cm^−1^) at 300 k 26. Despite these initial results, there has been no systematic study on the effect of Cs concentration or compositionally induced microstructural changes on the impedance measurement or ionic mobility. In this work, we measured the impedance of Ga-doped hollandite with varying Cs content in order to relate changes in conductivity to ionic mobility. Conductivity-temperature dependence for ionic conductivity based on an ion hopping mechanism can be described by the Arrhenius expression^27^


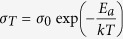


where σ_0_ is a preexponential factor proportional to the number of carrier ions; E_a_ is the activation energy in eV; k is the Boltzmann constant in 8.617 × 10^−5^ eV/K; and T is the absolute temperature in K. The activation energy in our study was calculated based on the total resistance from the low frequency intercept of the Nyquist plot from simulation results. Figure 4 shows Arrhenius plots of the conductivity values measured for hollandite with different Cs dopant level, indicating that the hollandite with the highest Cs concentration was the most conductive. Correspondingly, the activation energy of the Cs-containing sample (0.62 eV for Ba_0.66_Cs_0.66_ and 0.78 eV for Cs_1.33_) was smaller than that of the Ba-hollandite (0.99 eV for Ba_1.33_)as shown in Table 3. The existence of an impurity phase may also lead to a lower activation energy^28^. Compared to hollandite materials used in superionic conductor applications, such as K_1.6_Mg_0.77_Ti_7.23_O_16_, which has an activation energy of 0.23 eV^29^, the Ga-doped hollandite waste form has a relatively large activation energy with a corresponding large barrier to ionic transport. In addition, multiple arcs were observed in the Ba1.33 sample, which allowed the separation of bulk versus grain boundary conductivity (Fig. 4a). Of particular interest is the behavior of the end-member compositions. The bulk conductivity of the end-member composition Ba1.33 is similar in value to the total conductivity of the cesium end-member composition Cs1.33.

## Discussion

The mobility of Ba/Cs in the material is affected by many factors including the atomic structure and the microstructure. From the atomic perspective, the behavior is complicated because the ions in the tunnel interact not only with the framework octahedrons (from nearest oxygen to the cations in the octahedrons) but also with other tunnel ions. From the microstructural perspective, interfaces in the material, such as grain boundaries, also affect the conductivity on the macroscopic scale^30^. In the present work, since increased Cs content modifies both the atomic structure as well as the microstructure, the contribution of both effects are considered.

An important structural factor that limits the mobility of the Ba/Cs is the diagonal O1-O1 box shown in Fig. 1, which forms a bottleneck for the movement of Ba/Cs^25^. The Ba/Cs-O1 distance largely affects the diagonal box dimensions^31^. Barium and cesium cannot be distinguished using X-ray diffraction with copper radiation, and therefore the electronic density in the tunnel is assumed to correspond to a generic A-site species. As a result, the XRD refinement results are an average of Ba-O1 and Cs-O1 distances (A-O1). Comparing these three samples with different Cs concentration, we found that as Cs concentration increases, the average distance from tunnel ions to the nearest oxygen (A-O1) increases (Table 4). The refinement results compare well with DFT calculations, which also show an increased average Ba/Cs-O1 distance with increased Cs concentration. The increase of the lattice parameter is attributed to the increase of the A-O1 distance. Both the XRD refinement and DFT calculations showed the same lattice parameter trends. Discrepancies between the Ba-O1 distance and Cs-O1 distance may be attributed to both electronic interaction and ionic radius differences. The measured O1-O1 distances (5.07 to 5.50 Å) from DFT calculations are also listed in Table 4. The sum of the Ba and O ionic diameters is 5.64 Å; the sum of Cs and O ionic diameters is 6.28 Å. Therefore, the O1-O1 diagonal is small enough to suppress significant ion mobility in the hollandite structure.

**Figure 5:**
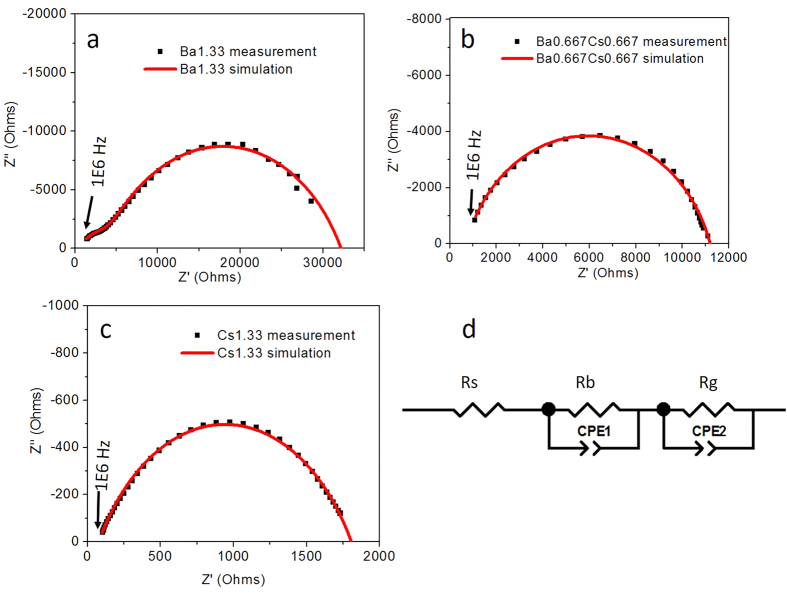
Nyquist plots and simulation results of (**a**) Ba1.33; (**b**) Ba0.667Cs0.667; (**c**) Cs1.33 at 800 °C; (**d**) General equivalent circuit used for simulation.

In addition to the atomic structure, microstructure also plays an important role on ionic conductivity. Nyquist plots overlaid with equivalent circuit modelling results for Ba1.33, Ba0.667Cs0.667, Cs1.33 are presented in Fig. 5. At high temperature (800 °C), Ba1.33 exhibited two distinguishable semicircles and were attributed to the bulk resistivity (R_g_) and grain boundary (R_gb_) resistivity, as shown in the equivalent circuit in Fig. 5d. However, the samples with the intermediate and highest concentration of Cs only exhibited a single semicircle attributed to the total conductivity of both the bulk and grain boundary contributions (Fig. 5b,c, respectively). Several studies have demonstrated that the growth direction of hollandite rod-like features is along [001], which is the tunnel direction of the hollandite crystal structure^32^,^33^,^34^. Figure 4b illustrates how the conductivity and microstructure of the studied hollandite vary with Cs concentration. An increase in both the ring size and rod length with increasing Cs concentration was observed. Considering the one-dimensional transport of Ba/Cs in the tunnel, a longer rod or larger ring size would be expected to increase tunnel ion transport. Based on the microstructure, it would appear that the total conductivity of Cs1.33 was greater than other compositions because it exhibited fewer grain boundary interfaces to disrupt ionic transport. In light of these microstructural effects, the total conductivity of Cs1.33 can be considered as essentially the bulk conductivity, which is similar in value and temperature dependence (activation energy) to the Ba1.33 end member. This agreement reflects the atomistic-scale structural similarities of the oxygen tunnel and O1-O1 diagonal, which effectively limits the mobility of large cations like Ba^2+^ and Cs^+^ in the hollandite structure. While limited in nature, there is motion of tunnel cations that could lead to ion migration and ultimately elemental release. The observation of grain size dependent total conductivity points to additional work that is needed to in order to understand elemental release and degradation as a function of different microstructural features currently encountered in waste form systems including melt processing (larger grain) versus solid state sintering processes (conventional sintering, hot press resulting in smaller grain size).

In summary, single-phase Ga-doped hollandite with varying Cs content has been successfully synthesized by a solid oxide reaction method. SEM and XRD confirmed the single-phase formation and homogeneous elemental distribution. DFT results predicted favorable phase formation for all of the compositions investigated with high Cs concentrations representing the most thermodynamically favorable. The bulk conductivity determined from AC impedance analysis of the Ba1.33 and Cs1.33 end members were found to have similar values and activation energy, which reflects the atomistic scale structural features that effectively limit the mobility of large cations like Ba^2+^ and Cs^+^ in the hollandite structure. The total conductivity was found to be dependent on microstructural features, with the high Cs-content compositions exhibiting large rod-like grains and the largest measured conductivity. From a comparison of atomic structural features and microstructure dependent property characterization, microstructure differences are likely to be the dominant factor that determines Ba and Cs ion mobility in hollandite. This observation points to the additional focus required to understand elemental release and degradation in hollandite as a function of composition and processing induced microstructural features.

## Methods

### Density Functional Theory Calculations

Density Functional Theory (DFT) calculations were conducted to examine the atomic structure and thermostability of the Ga-hollandite solid solution series. In order to have integer values for the number of atoms at the available A sites, 4 atoms were placed in 1 × 1 × 3 supercell resulting in a fixed A site occupancy of 4/6, or equivalently 1.33/2 for the 1 × 1 × 1 unit cell. Three compositions were selected by varying the proportion of Ba to Cs atoms on the A-site while maintaining fixed occupancy. The calculated compositions for the 1 × 1 × 3 supercell were: Ba_4_Ga_8_Ti_16_O_48_, Ba_2_Cs_2_Ga_6_Ti_18_O_48_, Cs_4_Ga_4_Ti_20_O_48_ corresponding to Ba1.33, Ba0.667Cs0.667, Cs1.33, respectively. The generalized gradient approximation with the Perdew-Burke-Ernzerhoff functional (GGA-PBE)^35^, as implemented in Cambridge Serial Total Energy Package (CASTEP)^36^, was used to approximate the electron exchange correlation energy. Ultrasoft pseudopotentials were used to approximate the behavior of the core electrons such that only the Ba 5s^2^ 5p^6^ 6s^2^, Cs 5s^2^ 5p^6^ 6s^1^, Ga3d^10^ 4s^2^ 4p^1^, Ti 3s^2^ 3p^6^ 3d^2^ 4s^2^, and O 2s^2^ 2p^4^ valence electrons were treated explicitly. Geometry optimizations were performed to an electronic tolerance of 2 × 10^−5^ eV/atom and a geometry tolerance of 2 × 10^−6^ eV/atom. In addition to the optimized structural properties (*i.e.*, unit cell parameters and interatomic spacing), the enthalpies of formation of the synthesized hollandite phases were calculated based on the reaction from the primary oxide components. For example, Reaction 1 describes the formation of Ba1.33 from the primary oxide components. The reaction enthalpy (*i.e.*, difference in the energy of the products and reactants) is the enthalpy of formation. The final energies from DFT calculations for each component of the reaction are used for the enthalpy of formation calculation. DFT calculations are ground state calculations at 0 K; therefore, the total energy of the calculation is essentially an enthalpy.


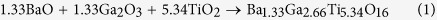


### Synthesis of hollandite

(Ba,Cs,Ga)-hollandite with the formula Ba_x_Cs_y_Ga_2x+y_Ti_8−2x−y_O_16_ (x = 1.33, 1.04, 0.667, 0; y = 0, 0.24, 0.667, 1.33) was prepared by solid-state reaction from reagent-grade oxide and carbonate powders: Ba_2_CO_3_, Ga_2_O_3_, TiO_2_, Cs_2_CO_3_. The baseline composition (x = 0) was prepared for reference^14^. Approximately 20 grams of dry oxide and carbonate precursors were added in stoichiometric proportions, mixed through ball milling, ground, and pressed into 13 mm diameter pellets with a force of 1300 psi. Pellets were calcined in air for 30 hours at 1150 °C. The calcined pellets were crushed, ball milled and re-pressed into pellets followed by sintering in air for 3 hours at 1250 °C. The sintered samples were used for subsequent characterization measurements. To reduce the risk of Cs vaporization during synthesis, the alumina crucible was sealed with alumina cement during both the calcination and sintering process.

### Characterization of Synthesized Hollandite

Inductively Coupled Plasma-Atomic Emission Spectroscopy (ICP-AES) was used to measure Ba, Ga, and Ti concentrations and Inductively Coupled Plasma-Mass Spectroscopy (ICP-MS) was used to measure Cs concentrations as Cs cannot be measured by ICP-AES. A representative amount from each sample was prepared via a sodium peroxide and lithium-metaborate fusion method for cation measurements. Prepared samples were analyzed twice for each element of interest by ICP, with the instrumentation being re-calibrated between the duplicate analyses. Standards were also intermittently measured to ensure the performance of the ICP instruments over the course of the analyses. The measured cation concentrations were converted to their respective and most stable oxide to obtain a wt. % of each component oxide. Phase identification was performed on samples by X-ray diffraction (XRD) with Cu Kα (λ = 1.54 Å) radiation (Bruker-AXS D8 Focus). Stepped scans from 20–70 deg. two-theta with 0.1 step increments for 3 seconds per step were used. The densities of sintered samples were measured by Archimedes method in deionized water. The relative density (ρ_a_/ρ_t_), where ρ_a_ is the density measured by Archimedes methods and ρ_a_ is the density calculated from XRD determined lattice parameter and molecular mass of the crystal. Rietveld refinement was performed with the GSAS software package^37^. Microstructure and phase composition were investigated using scanning electron microscopy (SEM) equipped with energy dispersive X-ray analysis (EDX) (Hitachi SU6600). Electrical properties were measured with a two-probe impedance method sweeping the frequency range 10^2^–10^7^ Hz (Solartron 1287 impedance/gain phase analyser). Measurements were made on cylindrical pellets 13 mm diameter and 1–2 mm thick. Platinum paste electrodes were applied to both sides of the pellets and impedance measurements were carried out in the temperature range 873–1073 K in air atmosphere.

## Additional Information

**How to cite this article**: Xu, Y. *et al.* A-site compositional effects in Ga-doped hollandite materials of the form Ba_x_Cs_y_Ga_2x+y_Ti_8−2x−y_O_16_: implications for Cs immobilization in crystalline ceramic waste forms. *Sci. Rep.* **6**, 27412; doi: 10.1038/srep27412 (2016).

## Figures and Tables

**Table 1:** Calculated (CASTEP calculations with GGA-PBE and ultra-soft pseudopotentials) enthalpies of the selected compositions.

Compositions	Formation Enthalpy (kJ/mol)
Ba_1.33_Ga_2.66_Ti_5.34_O_16_	−109.6
Ba_0.667_Cs_0.667_Ga_2_Ti_6_O_16_	−133.9
Cs_1.33_Ga_1.33_Ti_6.67_O_16_	−140.8

**Table 2:** Target and measured hollandite compositions based on semi-quantitative measurement by SEM-EDX (normalized to Ti) and ICP-MS and density ρ of sintered pellets.

Target composition	EDX composition	ICP-MS composition	ρ_a_	ρ_t_	ρ_a_/ρ_t_
Ba_1.33_Cs_0_Ga_2.66_Ti_5.34_O_16_	Ba_1.39_Cs_0_Ga_2.6_Ti_5.4_O_16_	Ba_1.3_Ga_2.7_Ti_5.3_O_16.0_	4.42	4.59	0.91
Ba_1.04_Cs_0.24_Ga_2.32_Ti_5.68_O_16_	Ba_1.09_Cs_0.229_Ga_2.6_Ti_5.68_O_16_	Ba_1.1_Cs_0.2_Ga_2.4_Ti_5.7_O_16.0_	4.31	4.74	0.91
Ba_0.667_Cs_0.667_Ga_2_Ti_6_O_16_	Ba_0.77_Cs_0.529_Ga_2.06_Ti_6_O_16_	Ba_0.7_Cs_0.5_Ga_2.1_Ti_6.0_O_16.1_	4.15	4.74	0.87
Ba_0_Cs_1.33_Ga_2.66_Ti_5.34_O_16_	Ba_0_Cs_1.22_Ga_1.44_Ti_6.67_O_16_	Ba_0_Cs_1.2_Ga_1.4_Ti_6.6_O_16.0_	3.92	4.66	0.84

**Table 3:** Activation energies determined from the temperature dependent conductivity.

Compositions	Ba_1.33_	Ba_0.66_Cs_0.66_	Cs_1.33_
Ea (eV)	0.99	0.62	0.78

**Table 4:** XRD refinement and DFT results showing key bond distances and lattice parameters.

Property	Composition	Calculated	Measured	Δ%
Average Ba/Cs-O1 (Å)	Ba_1.33_	2.76	2.872(6)	−3.8%
	Ba_0.667_Cs_0.667_	2.85	2.943(4)	−3.1%
	Cs_1.33_	2.98	3.120(1)	−4.5%
Average Ti/Ga-O(Å)	Ba_1.33_	0.19	0.194(5)	−2.1%
	Ba_0.667_Cs_0.667_	0.20	0.190(3)	5.3%
	Cs_1.33_	0.20	0.190(12)	5.3%
Average O1- O1 (Å)	Ba_1.33_	5.07		
	Ba_0.667_Cs_0.667_	5.43		
	Cs_1.33_	5.50		
a (Å)	Ba_1.33_	10.12	10.04	0.8%
	Ba_0.667_Cs_0.667_	10.26	10.14	1.2%
	Cs_1.33_	10.45	10.26	1.9%
c (Å)	Ba_1.33_	2.96	2.964	−0.1%
	Ba_0.667_Cs_0.667_	2.96	2.959	0.0%
	Cs_1.33_	2.96	2.959	0.0%

The difference between measured and calculated values are represented as a percent change (**Δ%**). The experimental uncertainties are in parentheses.
